# Impact of GLP-1 receptor agonist versus omega-3 fatty acids supplement on obesity-induced alterations of mitochondrial respiration

**DOI:** 10.3389/fendo.2023.1098391

**Published:** 2023-03-23

**Authors:** Kirsten M. Jansen, Norma Dahdah, Pau Gama-Perez, Pauke C. Schots, Terje S. Larsen, Pablo M. Garcia-Roves

**Affiliations:** ^1^Cardiovascular Research Group, Department of Medical Biology, Faculty of Health Sciences, UiT The Arctic University of Norway, Tromsø, Norway; ^2^Department Physiological Sciences, Faculty of Medicine and Health Sciences, University of Barcelona and Bellvitge Biomedical Research Institute (IDIBELL), Hospitalet del Llobregat, Spain

**Keywords:** mitochondrial dysfunction, lipotoxicity, obesity, GLP-1 receptor agonist, omega-3 fatty acid supplementation

## Abstract

**Objective:**

To compare administration of the glucagon-like peptide-1 (GLP-1) analogue, exenatide, versus dietary supplementation with the omega-3 fatty acid-rich Calanus oil on obesity-induced alterations in mitochondrial respiration.

**Methods:**

Six-week-old female C57BL/6JOlaHSD mice were given high fat diet (HFD, 45% energy from fat) for 12 weeks to induce obesity. Thereafter, they were divided in three groups where one received exenatide (10 μg/kg/day) via subcutaneously implanted mini-osmotic pumps, a second group received 2% Calanus oil as dietary supplement, while the third group received HFD without any treatment. Animals were sacrificed after 8 weeks of treatment and tissues (skeletal muscle, liver, and white adipose tissue) were collected for measurement of mitochondrial respiratory activity by high-resolution respirometry, using an Oroboros Oxygraph-2k (Oroboros instruments, Innsbruck, Austria).

**Results:**

It was found that high-fat feeding led to a marked reduction of mitochondrial respiration in adipose tissue during all three states investigated – LEAK, OXPHOS and ETS. This response was to some extent attenuated by exenatide treatment, but not with Calanus oil treatment. High-fat feeding had no major effect on hepatic mitochondrial respiration, but exenatide treatment resulted in a significant increase in the various respiratory states in liver. Mitochondrial respiration in skeletal muscle was not significantly influenced by high-fat diet or any of the treatments. The precise evaluation of mitochondrial respiration considering absolute oxygen flux and ratios to assess flux control efficiency avoided misinterpretation of the results.

**Conclusions:**

Exenatide increased hepatic mitochondrial respiration in high-fat fed mice, but no clear beneficial effect was observed in skeletal muscle or fat tissue. Calanus oil did not negatively affect respiratory activity in these tissues, which maintains its potential as a dietary supplement, due to its previously reported benefits on cardiac function

## 1 Introduction

In obesity and insulin resistance, impaired suppression of adipose tissue lipolysis contributes to elevated plasma levels and uptake of fatty acids in tissues like skeletal muscle, liver and heart which exceeds the mitochondrial oxidation capacity (1–4). As a result, there is an increased production of reactive oxygen species (ROS) with the accumulation of toxic lipid intermediates, which leads to mitochondrial dysfunction (5, 6). These alterations further aggravate the state of insulin resistance and promote the development of type 2 diabetes mellitus. The described conditions and associated co-morbidities represent major health issues (7), and the need for preventive strategies is therefore urgent.

Glucagon-like peptide-1 (GLP-1) is a polypeptide incretin hormone secreted by the L‐cells of the gastrointestinal tract in response to dietary signaling. Binding to and activation of the GLP-1 receptor enhances glucose-dependent insulin secretion, delays gastric emptying and suppresses appetite (8). Therefore, GLP-1 has been regarded as an interesting target in the treatment of obesity and type 2 diabetes. However, the short half‐life of native GLP‐1 in the circulation (< 2 minutes) limits its therapeutic potential, and for this reason several synthetic GLP-1 receptor agonists (incretin mimetics or GLP-1 analogues) have been introduced in the clinic (8). Exenatide is an FDA-approved drug in the incretin mimetic class and is indicated for treatment of type 2 diabetes mellitus and obesity when used in combination with diet and exercise (9). Furthermore, clinical trials have demonstrated that such GLP-1 analogues have the ability to induce weight loss (10, 11) and prevent development of hepatic steatosis (12). However, little is known about the effect of exenatide treatment on mitochondrial respiration.

Dietary supplementation with marine omega-3 fatty acids is considered to have beneficial health effects, due to its lipid-lowering effect (13, 14), and treatment of severely obese non-diabetic patients with eicosapentaenoic acid and docosahexaenoic acid was shown to reduce adipose tissue mass (14). Calanus oil is a novel marine oil, extracted from the marine crustacean, Calanus finmarchicus (15). It is rich in and mono- and polyunsaturated omega-3 fatty acids (especially stearidonic acid) and used as a dietary supplement in humans. However, results regarding the effects of omega-3 fatty acids on glucose metabolism, insulin resistance, and type 2 diabetes are still controversial (16), most likely due to differences in the choice of preparation, dosage, and intervention.

In a recent study, we showed that administration of exenatide reduced deposition of intra-abdominal fat, and at the same time improved the capacity for myocardial glucose oxidation during high-fat feeding in mice (17). Similar results were obtained by dietary supplementation with small amounts (2%) of Calanus oil. Thus, the purpose of this study was to examine and compare the impact of dietary Calanus oil versus exenatide administration on mitochondrial respiration in major tissues, such as adipose tissue, liver, and skeletal muscle from high-fat fed mice.

## 2 Materials and methods

### 2.1 Animals

Female C57BL/6JOlaHSD mice were purchased from Envigo (Indiana, IN, USA) at 5 weeks of age. The animals were housed at 23 °C, three animals per cage, under a reversed light/dark cycle (12-h dark/12-light). At the age of 6 weeks, mice were split into two groups: the control group fed a normal chow diet (NCD, n=10) containing 10% energy from fat (#58Y2, Test Diet, IPS Ltd, Notts, UK) and the other group fed a high-fat diet (HFD, 45% energy from fat) (#58V8, Test Diet, IPS Ltd, Notts, UK). Mice from all groups were fed *ad libitum*. After 12 weeks, the HFD-fed mice were split into three subgroups (each consisting of 10 mice) – one receiving HFD without any supplement (HFD), a second subgroup receiving HFD supplemented with 2% Calanus oil (HFD + Cal) which was mixed into the high-fat diet by the manufacturer (Test Diet; IPS, Notts, UK), while a third subgroup received 10 µg/kg/day of the incretin mimetic, exenatide (Polypeptide Laboratories Pvt Limited, Ambernath India), *via* mini-osmotic pumps (Alzet Micro-Osmotic Pump Model 1004, DURECT Corporation, ALZET Osmotic Pumps, Cupertino, CA, USA). This feeding regimen continued for another 8 wk, so that the total feeding period lasted 20 wk. The control group (NCD, n=10) received NCD throughout the whole feeding period. Specific n size per group and tissue are specified in each figure and table (the n size is reduced in some groups due to technical reasons, or samples lost)

*Pumps installation:* The first 3-4 days after surgery and insertion of mini-osmotic pumps, the mice were single-housed to secure healing of the operation wound. Furthermore, mice who did not undergo surgery were subjected to single housing as well. Temgesic analgesia (0.1 mg/kg) was given 8 and 20 hours postoperatively. A few mice were classified as low responders to the high fat diet (mice that did not increase their body weights above that of the lean controls) or high responders (mice whose body weight exceeded 40 g). These mice were excluded from the study.

All animal procedures were approved by the local ethics committee, Comitè Ètic d’Experimentació Animal at the Universitat de Barcelona and the Departament d’Agricultura, Ramaderia, Pesca, Alimentació i Medi Natural at the Generalitat de Catalunya, complying with current Spanish and European legislation.

### 2.2 Tissue collection and sample preparation for Oxygraph-2k

The mice were anesthetized with Avertin (2,2,2-tribromoethanol 99% and tertiary amyl alcohol [1:1 w/v], 500 mg/kg body weight) and tissues were collected in the following order: skeletal muscle *Extensor Digitorum Longus* (EDL) followed by gonadal white adipose tissue (WAT) and finally liver. Right after collection, EDL and liver were kept in biopsy preservation solution (BIOPS) while WAT was kept in mitochondrial respiration media MiR05 (for details about the buffers refer to (18).

*Tissue preparation for high-resolution respirometry:* Each tissue in this study was processed differently to optimize it for mitochondrial respiration assays. The major distinctions in tissue preparations were the following. For skeletal muscle EDL, muscle fibers were separated to facilitate the permeabilization by saponin. No chemical permeabilization was needed for liver while WAT was permeabilized with digitonin but both required mechanical permeabilization and homogenization by mechanical shredder (PBI shredder SG3, Oroboros Instruments) (protocols detailed in (18).

### 2.3 High-resolution respirometry

Mitochondrial function for EDL, liver and WAT was assessed by high-resolution respirometry (HRR) in Oxygraph-2k system (Oroboros instruments, Innsbruck, Austria). The experimental design for this assay was a substrate-uncoupler-inhibitor titration (SUIT-8, https://wiki.oroboros.at/index.php/SUIT-008) HRR protocol previously established (19). Through this protocol, mitochondrial respiration was measured at the level of multiple respiratory states defined briefly in the next section.

Firstly, in the presence of the NADH (N)-linked substrates Pyruvate (5 mM) and Malate (2 mM), LEAK respiration (non-phosphorylating resting state) was assessed. Then, by adding ADP (5 mM), oxidative phosphorylation (OXPHOS) capacity was determined and by adding cytochrome C (10 μM) the integrity of the mitochondrial outer membrane was assessed. Glutamate (10 mM) was later added to measure maximal capacity of the NADH pathway. Next, Succinate (10 mM) was added to stimulate the succinate (S)-linked pathway, allowing the convergent electron flow through both pathways simultaneously. Uncoupler carbonyl cyanide p-trifluoro-methoxyphenyl hydrazine (FCCP, 0.5 μM) was repeatedly titrated until maximal non-coupled Electron Transfer (ET)-respiratory capacity mediated by NS-linked pathways was reached. To determine the ET-respiratory capacity mediated uniquely by S-pathway, Complex I inhibitor Rotenone (0.5 μM) was added. And finally Complex III inhibitor Antimycin A (2.5 μM) was used to quantify residual oxygen consumption (ROX) which was subtracted from O_2_ flux as a baseline for all respiratory states. A significant increase in O_2_ flux after the addition of cytochrome c it will be a technical criterion to exclude the sample from our data analysis.

All substrates and inhibitors were added at saturated concentrations. The O2k-Software DatLab 7.4 was used for real-time data acquisition and analysis. Oxygen flux values were expressed relative to tissue wet weight per second (*JO_2_*, pmol mg-1 s-1). Flux control ratios were calculated by dividing the corresponding value by that of the maximal non-coupled Electron Transfer (ET)-respiratory capacity.

### 2.4 Statistical analysis

Data are presented as the means with their standard errors of the mean (SEM). Graphs and statistics are done in GraphPad Prism 8.4.2 (GraphPad Software, LLC). Significant differences between treatment groups were assessed by one-way ANOVA followed by Tukey’s *post-hoc* test where all groups were compared to each other.

## 3 Results

To determine the effect of Calanus oil and exenatide treatments on mitochondrial bioenergetics we have performed a detailed analysis of mitochondrial respiratory capacity under saturated conditions of substrates and ADP. We have studied, in detail, different respiratory states and flux control ratios (as defined by Gnaiger in Mitochondrial pathways and respiratory control: An introduction to OXPHOS analysis) (20). Those studies were performed in tissue homogenates preparations of liver, white adipose tissue, and skeletal muscle permeabilized fibers. Mice were treated with Calanus oil (dietary supplementation) or exenatide (via subcutaneous implanted mini-osmotic pumps) during the last 8 weeks of the experimental period. Food intake and body weight evolution are represented in **Tables 1** and **2**.

**Table 1:** Average food (and energy) intake during the 8 weeks treatment period.

	Food intake	Energy intake
Group	g/day	kJ/day
NCD	2.63 ± 0.03	41.61 ± 0.65*
HFD	2.57 ± 0.07	50.01 ± 1.29
HFD+Cal	2.79 ± 0.08	54.29 ± 1.54
HFD+Ex	2.37 ± 0.07	46.51 ± 1.29*

Data are given as mean ± SEM (n=10 in each group). NCD, normal chow diet; HFD, high-fat diet; HFD+Cal, high-fat diet supplemented with Calanus oil; HFD+Ex, high-fat diet combined with exenatide infusion. Energy intake was calculated based on the energy content of NCD (58Y2, Test Diet) and HFD diets (58V8, Test Diet) – 15.82 and 19.46 kJ/g, respectively. *, p<0.05 vs HFD.

**Table 2:** Average body weight at the start (wk 12) and end (wk 20) of the treatment period, as well as weight gain during the treatment period.

	Body weight wk 12	Body weight wk 20	Body weight gain
Group	g	g	g
NCD	23.7 ± 0.46	25.4 ± 0.53	1.71 ± 0.33*
HFD	28.3 ± 1.00	33.7 ± 0.81	5.42 ± 0.75
HFD+Cal	29.2 ± 0.96	36.3 ± 2.26	7.14 ± 0.72
HFD+Ex	27.9 ± 1.39	29.2 ± 0.71	1.65 ± 0.62*

Data are given as mean ± SEM (n=10 in each group). NCD, normal chow diet; HFD, high-fat diet; HFD+Cal, high-fat diet supplemented with Calanus oil; HFD+Ex, high-fat diet combined with exenatide infusion. *, p<0.05 vs HFD.

### 3.1 LEAK

First, we analyzed the effects of the high fat diet (HFD) and treatments on the LEAK state. *“The LEAK state is defined as a state of mitochondrial respiration when O_2_ flux mainly compensates for ion leaks in the absence of ATP synthesis, at kinetically-saturating concentrations of O_2_ and respiratory fuel substrates”* (20). *“The LEAK rate is a function of respiratory state, hence it depends on (1) the barrier function of the mitochondrial inner membrane (“leakiness”), (2) the electrochemical potential differences and the concentration differences across the mitochondrial inner membrane, and (3) the H^+^/O_2_ ratio of the Electron Transfer pathway.”* (20). Thus, LEAK respiration gives an estimation of intrinsic uncoupling in the absence of adenylates.

**Figure 1:**
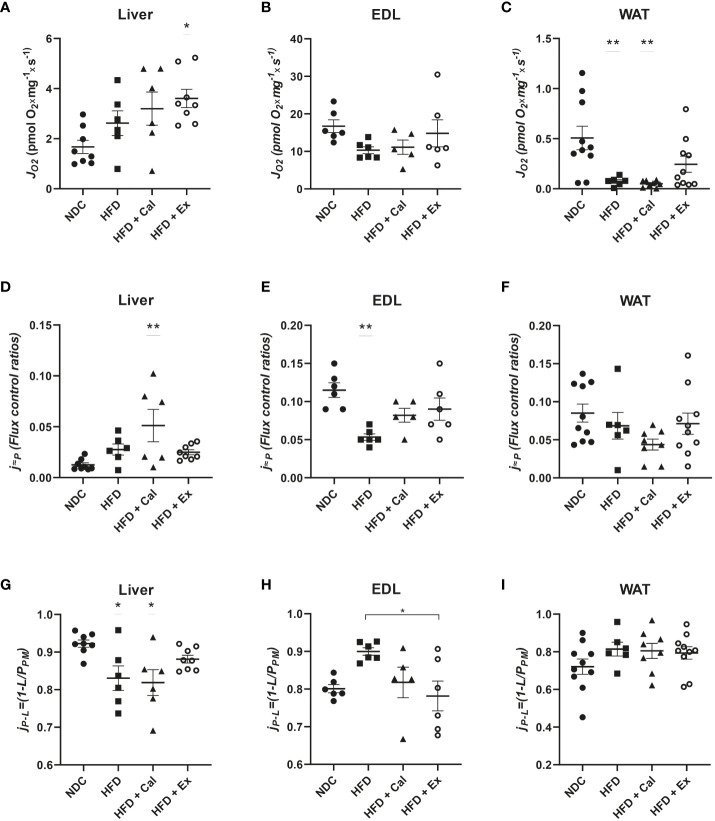
LEAK state respiration. Absolute oxygen flux (*J*O_2_) in the LEAK state in liver (n size NDC=8, HFD=6, HFD+cal=6, HFD+Ex=8) **(A)**, extensor digitorum longus (EDL) muscle (n size NDC=6, HFD=6, HFD+cal=5, HFD+Ex=6) **(B)** and white adipose tissue (WAT) (n size NDC=10, HFD=6, HFD+cal=8, HFD+Ex=10) **(C)** from mice given normal chow diet (NCD), high-fat diet (HFD), high-fat diet supplemented with Calanus oil (HFD+Cal) and high-fat diet combined with exenatide infusion (HFD+Ex). Oxygen flux in the LEAK state normalized to maximum oxygen flux (j≈P) for the same tissues is shown **(D–F)**, while ratios demonstrating flux control efficiency (j_P_-L=(1-L/P_PM_) are shown in **(G–I)** Data are represented as individual values and mean ± SEM, and differences between groups were analyzed by one-way analysis of variance (ANOVA) and *post-hoc* Tukey’s tests. *, p < 0.05; **, p < 0.01 compared to the NCD group unless otherwise indicated.

In our case this state was measured under saturated concentrations of malate and pyruvate. Liver absolute oxygen flux in the LEAK state was not affected by 20 weeks on a HFD when compared with the results obtained in lean control mice under a regular rodent chow diet. Similarly, calanus treatment did not show any effect at the LEAK state, but exenatide treatment showed a significant increase in oxygen flux with respect to the control experimental group (**Figure 1A**). Same analysis showed no significant differences at the LEAK state in EDL muscle pfi preparations (**Figure 1B**). Concerning WAT, there was a significant reduction of the oxygen flux at the LEAK state for the mice on a HFD and HFD treated with calanus (**Figure 1C**).

Considering differences in mitochondrial density, it is important to additionally assess LEAK through other relative parameters that contemplate those differences. The flux control ratios of the LEAK state normalized by the maximum oxygen flux (obtained when assessing maximal electron transfer system (ETS) capacity stimulated by the addition of the uncoupler FCCP) led to a different interpretation of the effect of the different treatments on LEAK. In liver, a significant increase was observed after calanus treatment with respect to control mice, with no difference among the other groups (**Figure 1D**). However, in EDL muscle 20 weeks of a HFD treatment significantly reduced the contribution of LEAK to maximal oxygen consumption, not reaching significance when the mice on HFD were treated with calanus or exenatide (**Figure 1E**). We did not find significant changes when the same flux control ratio was compared among the different experimental groups in WAT (**Figure 1F**).

Finally, we assessed whether LEAK affects NADH pathway net OXPHOS capacity available for ATP production by calculating flux control efficiency (*j_P-L_=1-L/P_PM_*) where *P_PM_* was the oxygen flux after subsequent addition of ADP and cytochrome c (pyruvate and malate were already added to evaluate the LEAK state) (**Figures 1G–I**). Thus, we observed in the liver that HFD and calanus treatment significantly reduced the capacity for ATP production after the addition of saturated concentrations of ADP. In EDL muscle we observed a different pattern, HFD tended to increase couple respiration which was significantly prevented/reverted by exenatide treatment. No net effect of LEAK on OXPHOS capacity for ATP production was seen in WAT.

### 3.2 OXPHOS state

After the evaluation of the LEAK state, we proceeded to evaluate the OXPHOS state with substrates feeding the NADH pathway. *“The OXPHOS state is defined as the respiratory state with kinetically-saturating concentrations of ADP and Pi (phosphorylation substrates), respiratory fuel substrates and O_2_ in the absence of exogenous uncoupler, to estimate the maximal respiratory capacity in the OXPHOS state for any given electron transfer pathway state (mainly, NADH, Succinate, electron transferring flavoprotein complex)”* (20). We first evaluated the effect of saturated concentrations of ADP and Cyt c in the presence of saturated concentrations of malate and pyruvate. Cyt c was added to assess the stability of the mitochondrial inner membrane. To address potential differences in pyruvate dehydrogenase complex capacity, subsequently we added saturated concentrations of glutamate to assess maximal capacity of the NADH pathway (Complex I) and observe if any potential differences in oxygen flux at the couple state, with pyruvate and malate as substrates, could be compensated by the addition of glutamate.

**Figure 2:**
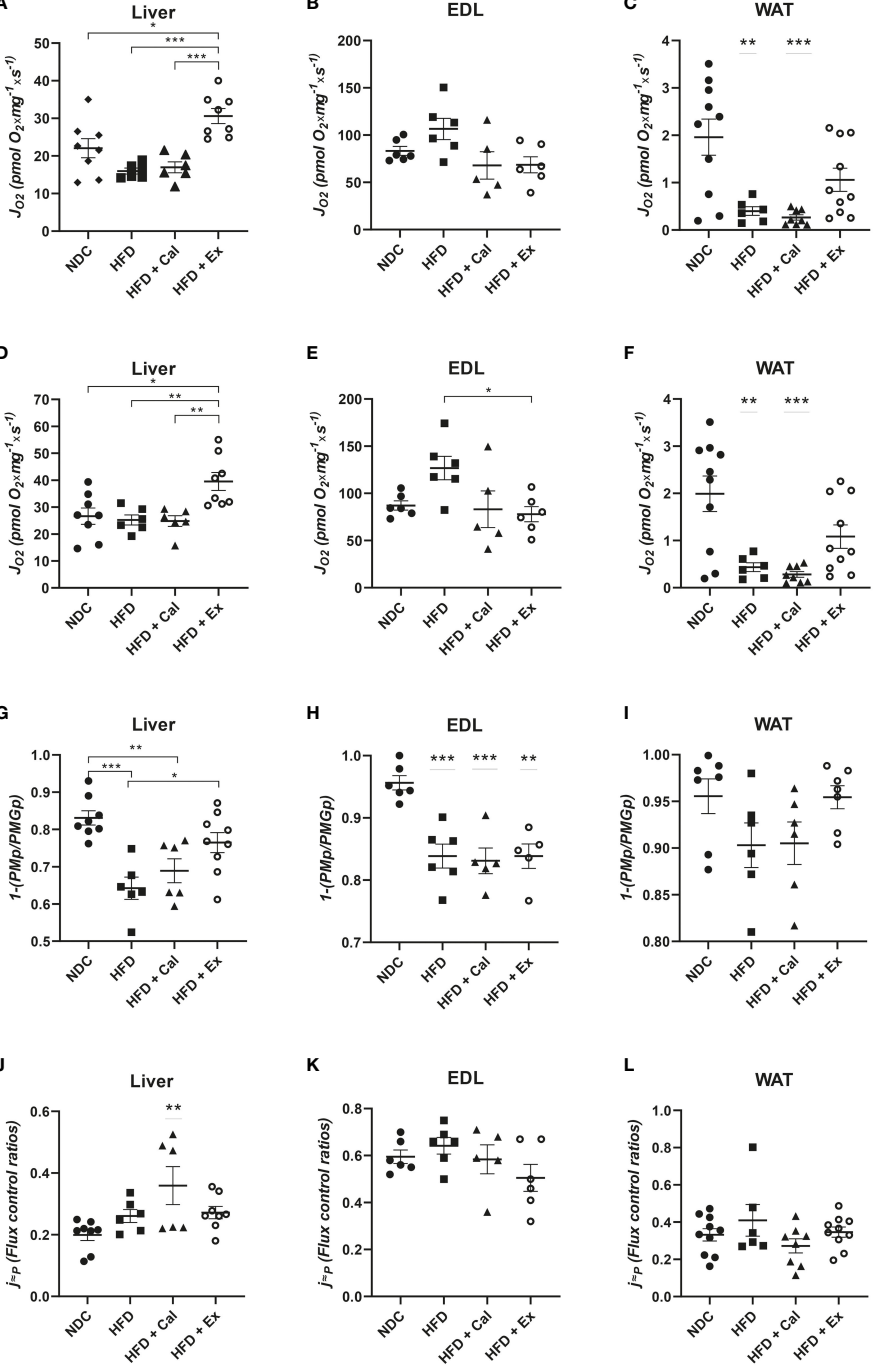
OXPHOS state – with focus on NADH pathway. Oxygen flux (*J*O_2_) in the OXPHOS state after ADP addition in liver (n size NDC=8, HFD=6, HFD+cal=6, HFD+Ex=8) **(A)**, extensor digitorum longus (EDL) muscle (n size NDC=6, HFD=6, HFD+cal=5, HFD+Ex=6) **(B)** and white adipose tissue (WAT) (n size NDC=10, HFD=6, HFD+cal=8, HFD+Ex=10) **(C)** from mice given normal chow diet (NCD), high-fat diet (HFD), high-fat diet supplemented with Calanus oil (HFD+Cal) and high-fat diet combined with exenatide infusion (HFD+Ex). Oxygen flux in the OXPHOS state after addition of glutamate is shown for the same tissues in **(D–F)**, while ratios demonstrating flux control efficiency of complex I in the OXPHOS state after the addition of glutamate (1-(PMp/PMGp) are shown in **(G–I)** Finally, oxygen flux in the OXPHOS state (contribution by the NADH pathway) normalized to maximum oxygen flux is shown in **(J, K)** and **(L)**. Data are represented as individual values and mean ± SEM, and differences between groups were analyzed by one-way analysis of variance (ANOVA) and *post-hoc* Tukey’s tests. *, p < 0.05; **, p < 0.01; ***, p < 0.001. In the absence of lines between groups, symbols of a statistically significant difference above a group are in comparison to the NCD group.

In liver, a similar pattern to that described at the LEAK state was found; treatment with exenatide in high-fat fed mice significantly increased oxygen flux (**Figure 2A**). No differences among groups were detected in EDL muscle at this OXPHOS state (**Figure 2B**). However, in WAT we measured a significant reduction in oxygen flux in mice fed with a HFD, not reverted by calanus treatment but partially reverted by exenatide treatment (not being significantly different from the NDC group) (**Figure 2C**).

Same differences among groups were measured after the addition of glutamate to assess maximal capacity of the NADH pathway, with the exception in skeletal muscle for the mice treated with exenatide (HFD+Ex) which showed a reduction in oxygen flux with respect to HFD untreated mice (**Figures 2D–F**).

To assess any potential difference in pyruvate dehydrogenase complex activity (supplying reductive potential to complex I from pyruvate) we calculated the flux control efficiency of complex I at the OXPHOS state after the addition of Glutamate (1-(*PMp/PMGp*)). Pyruvate and malate complex I substrates at saturated concentrations were able to almost maximize complex I activity (more than 83% in liver, and above 95% in EDL muscle and WAT) (**Figures 2G–I**). When mice were on a HFD this contribution was significantly reduced in liver and EDL muscle, presenting the same tendency in WAT but not reaching statistical significance.

In our experimental protocol maximal oxygen flux is reached by the convergent electron transfer from both NADH and succinate after the addition of the exogenous uncoupler FCCP (ETS capacity). Taking this into account, Complex I relative contribution to maximal oxygen flux under saturated concentrations of ADP in control mice, were 0.199 ± 0.018; 0.595 ± 0.028; and 0.332 ± 0.033 for liver, skeletal muscle, and WAT respectively. These results showed the dominance of the succinate pathway in liver and WAT, while in skeletal muscle NADH pathway played a more relevant role (**Figures 2J–L**) In liver NADH contribution to maximal oxygen flux is increased with the calanus treatment (**Figure 2J**).

**Figure 3:**
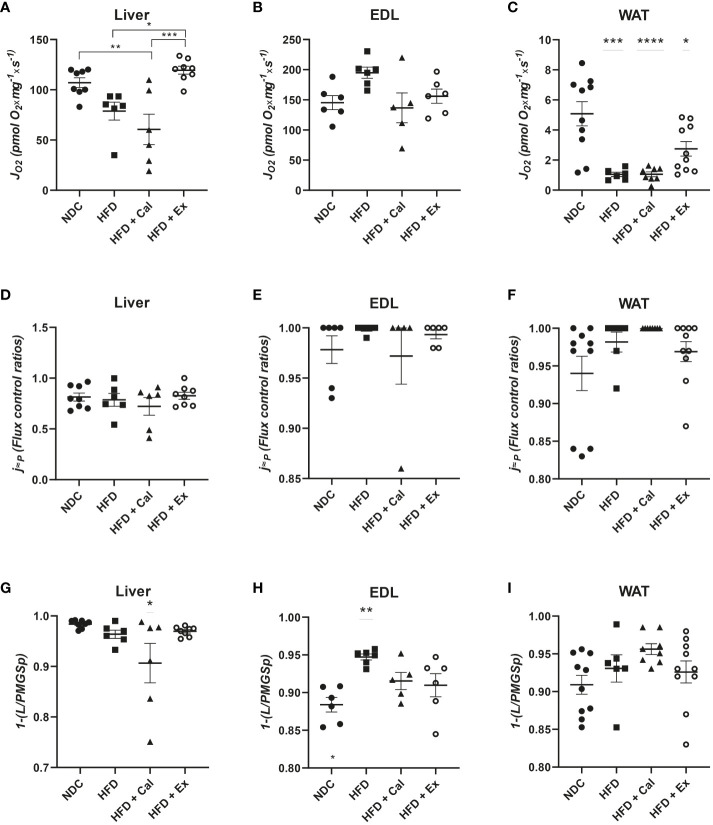
OXPHOS state in the 4 different experimental groups after the addition of succinate. Oxygen flux (*J*O_2_) in the OXPHOS state under convergent electron transfer from NADH and succinate dehydrogenase pathways in liver (n size NDC=8, HFD=6, HFD+cal=6, HFD+Ex=8) **(A)**, extensor digitorum longus (EDL) muscle (n size NDC=6, HFD=6, HFD+cal=5, HFD+Ex=6) **(B)** and white adipose tissue (WAT) (n size NDC=10, HFD=6, HFD+cal=8, HFD+Ex=10) **(C)** from mice given normal chow diet (NCD), high-fat diet (HFD), high-fat diet supplemented with Calanus oil (HFD+Cal) and high-fat diet combined with exenatide infusion (HFD+Ex). Oxygen flux in the OXPHOS state (convergent NADH and SDH pathways) normalized to maximum oxygen flux for the same tissues is shown in **(D, E)** and **(F)** Flux control efficiency (j≈P), i.e. ratio between OXPHOS (convergent NADH and SDH pathways) and LEAK respiration is shown in **(G, H)** and **(I)** Data are represented as individual values and mean ± SEM, and differences between groups were analyzed by one-way analysis of variance (ANOVA) and *post-hoc* Tukey’s tests. *, p < 0.05; **, p < 0.01; ***, p < 0.001; **** p, < 0.0001. In the absence of lines between groups, symbols of a statistically significant difference above a group are in comparison to the NCD group.

Using saturating substrates concentrations and convergent electron transfer pathways to provide reductive potential to CoQ10 allowed the evaluation of the upper limits of OXPHOS performance. Thus, next we evaluated OXPHOS oxygen flux after the subsequent addition of saturated concentrations of succinate, maximizing the activity of the NADH and succinate dehydrogenase pathways. The treatment with calanus reduced maximal OXPHOS capacity when compared to livers from control mice. However, exenatide treatment significantly reestablished oxygen flux values to control values when compared to HFD and HFD + Cal mice (**Figure 3A**). We did not find any differences when same statistical analysis was performed in skeletal muscle (**Figure 3B**). In WAT, HFD induced a significant reduction in maximal OXPHOS capacity unable to be rescued by any of the treatments (**Figure 3C**).

To complete our analysis of the effect of HFD and subsequent calanus or exenatide treatment on OXPHOS respiratory state we calculated the flux control ratio between maximal OXPHOS capacity and maximal ETS capacity (after the addition of FCCP). This ratio provides information related to control by coupling and the potential limitations of the phosphorylation system (20). Thus, we observed that in mice both skeletal muscle and WAT are very much coupled not being limited by the phosphorylation system at maximal electron transfer capacity (flux control ratios near 1 or 1) (**Figures 3E, F**). No differences were observed among experimental groups of EDL muscle and WAT. However, in liver coupling efficiency is close to 80% of maximal electron transfer capacity, which is not affected by HFD and subsequent calanus or exenatide treatment (**Figure 3D**).

One additional piece of information came from the evaluation of the net OXPHOS capacity available for ATP production by calculating OXPHOS (P)-LEAK (L) control efficiency (1-L/P). Calanus treatment reduces net OXPHOS capacity in liver (**Figure 3G**) and HFD increased its capacity in skeletal muscle, which is prevented by calanus or exenatide treatments (**Figure 3H**). Net OXPHOS capacity was unaffected by any experimental condition in WAT (**Figure 3I**).

### 3.3 ETS

**Figure 4:**
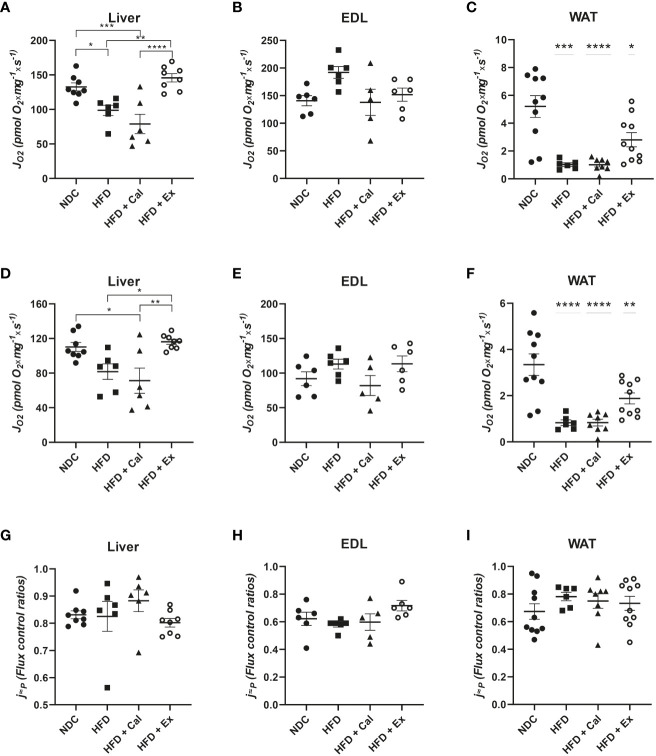
ETS state in the 4 different experimental groups. Oxygen flux (JO_2_) in the ETS state under convergent electron transfer from NADH and succinate dehydrogenase pathways in liver (n size NDC=8, HFD=6, HFD+cal=6, HFD+Ex=8) **(A)**, extensor digitorum longus (EDL) muscle (n size NDC=6, HFD=6, HFD+cal=5, HFD+Ex=6) **(B)** and white adipose tissue (WAT) (n size NDC=10, HFD=6, HFD+cal=8, HFD+Ex=10) **(C)** from mice given normal chow diet (NCD), high-fat diet (HFD), high-fat diet supplemented with Calanus oil (HFD+Cal) and high-fat diet combined with exenatide infusion (HFD+Ex). Oxygen flux in the ETS state under the succinate dehydrogenase pathway for the same tissues is shown **(D, E)** and **(F)** Flux control efficiency (j≈P), i.e. ratio between oxygen flux in the ETS state (convergent NADH and SDH pathways) and and LEAK respiration is shown in **(G, H)** and **(I)** Data are represented as individual values and mean ± SEM, and differences between groups were analyzed by one-way analysis of variance (ANOVA) and *post-hoc* Tukey’s tests. *, p < 0.05; **, p < 0.01; ***, p < 0.001; **** p, < 0.0001. In the absence of lines between groups, symbols of a statistically significant difference above a group are in comparison to the NCD group.

To assess electron transfer (ET) system capacity under the convergent action of the NADH and succinate pathways it is necessary to uncouple electron transfer from the capacity of the ATP synthase to produce ATP using exogenous uncouplers. *“The ET state is defined as the noncoupled state with optimum exogenous uncoupler concentration for maximum O_2_ flux at kinetically-saturating concentrations of respiratory fuel substrates and O_2_.”* (20). Uncouplers are weak lipid-soluble acids which function as protonophores and in this study we used carbonyl cyanide p-trifluoromethoxyphenylhydrazone (FCCP) for this purpose. In liver we observed similar differences among groups to those observed under the OXPHOS state, HFD and HFD mice treated with calanus oil showed a significant reduction in their oxygen flux at their maximal ET capacity; exenatide treatment was able to prevent this decrease, presenting similar oxygen fluxes than those of control mice (**Figure 4A**) A similar pattern is observed in WAT but with a more accentuated decrease in oxygen flux in the HFD and HFD calanus treated groups; in this tissue exenatide treatment was not able to restore oxygen flux to the same values reported in the control group (**Figure 4C**). In skeletal muscle oxygen fluxes at maximal ET capacity were similar among experimental groups (**Figure 4B**).

We inhibited NADH pathway by the addition of the complex I inhibitor rotenone at the ET state, which allowed us to evaluate the specific contribution of the succinate pathway. Oxygen fluxes in liver, EDL muscle and WAT presented similar patterns than when both pathways were acting in convergence (**Figures 4D–F**).

**Figure 5:**
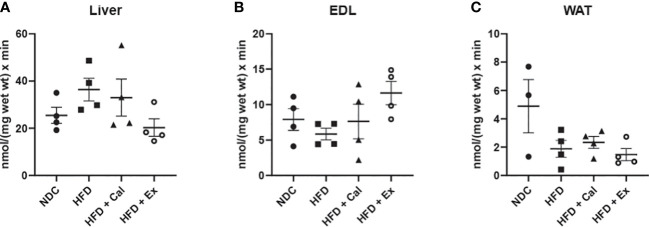
Citrate synthase activity. Citrate synthase (CS) activity of liver (n size NDC=4, HFD=4, HFD+cal=4, HFD+Ex=4) **(A)**, extensor digitorum longus (EDL) muscle (n size NDC=4, HFD=4, HFD+cal=4, HFD+Ex=4) **(B)** and white adipose tissue (WAT) (n size NDC=3, HFD=4, HFD+cal=4, HFD+Ex=4) **(C)** from mice given normal chow diet (NCD), high-fat diet (HFD), high-fat diet supplemented with Calanus oil (HFD+Cal) and high-fat diet combined with exenatide infusion (HFD+Ex). Data are represented as individual values and mean ± SEM. No statistically differences were observed between the groups for any of the tissues.

However, the relative contribution of the succinate pathway at the ET state to maximal oxygen flux was the same in the three tissues under study, indicating that absolute oxygen fluxes at this specific state were more indicative of the differences in mitochondrial density than to qualitative changes in the electron transfer system (**Figures 4G–I**). However, citrate synthase activity (a marker of mitochondrial content) do not showed significant differences among experimental conditions (**Figure 5**) but in WAT could be limited by the number of samples and variability observed.

### 3.4 Summary of mitochondrial respiration per tissue and condition

**Figure 6:**
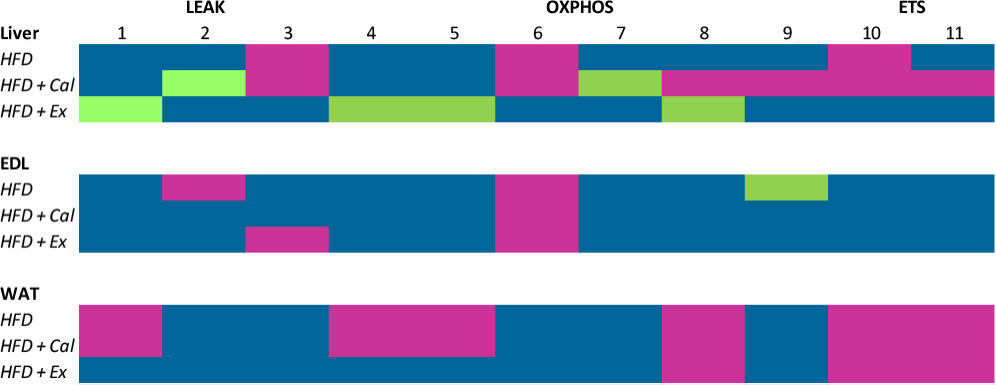
Summary of mitochondrial respiration per tissue and condition. Fluxes and ratios demonstrated in the previous graphs are put together, illustrating the changes in the various tissues from mice given high-fat diet (HFD), high-fat diet supplemented with Calanus oil (HFD+Cal) and high-fat diet combined with exenatide infusion (HFD+Ex) compared to the normal control group. The numbers represent the following; 1: Malate and pyruvate, 2: Malate and pyruvate flux control rate, 3: Flux control efficiency, 4: Malate and pyruvate in the presence of saturated concentrations of ADP, 5: 4+ glutamate, 6 Maximum complex I activity, 7: Complex I relative contribution to maximal oxygen flux, 8: OXPHOS oxygen flux after the subsequent addition of saturated concentrations of succinate, 9: Net OXPHOS capacity available for ATP production, OXPHOS (P)-LEAK (L) control efficiency (1-L/P), 10: OXPHOS state + uncoupler, 11: 10+ complex I inhibitor rotenone. Color codes are: Blue, no significant difference; purple, significant reduction; green, significant increase.

**Figure 6** simplifies treatment effect on mitochondrial respiration when compared to values registered in the liver, EDL and WAT of control mice. In liver, HFD reduced mitochondria respiratory markers in the three respiratory states analyzed (LEAK, OXPHOS and ETS), which was not prevented by calanus treatment but did by exenatide treatment. The impact of the HFD as well as that of the additional treatments with calanus oil or exenatide was not major on skeletal muscle mitochondrial respiration (EDL muscle). Being maximal NADH pathway oxygen, consumption reduced after HFD or HFD+calanus or HFD+exenatide. In WAT tissue of the HFD group, there was a significant decrease in mitochondrial oxygen consumption at the three respiratory states, which was not prevented by co-treatment with calanus oil and was only partially prevented by the co-treatment with exenatide (LEAK and OXPHOS states).

In summary, results from liver data bring into light the potential positive impact of exenatide treatment on liver mitochondrial respiratory capacity. Moreover, and as important, none of the treatments (calanus oil or exenatide) showed a deleterious effect accentuating the reduction of mitochondrial respiratory capacity due to HFD feeding in all three tissues under study making these treatments, even though not necessarily beneficial for the different mitochondrial respiration states in all tissues, not harmful either.

## 4 Discussion

Previous studies have reported that human obesity and high-fat diet-induced obesity in rodents are associated with mitochondrial dysfunction, mostly attributed to an imbalance between fatty acid supply and oxidation (21–25). Glucagon-like peptide 1 (GLP-1) analogues or incretin mimetics are commonly used as anti-obesogenic or anti-diabetic drugs (26, 27), and the GLP-1 analogue, exenatide, has been shown to prevent or attenuate obesity-induced mitochondrial dysfunction in the heart (28). Non-pharmacological treatment of obesity includes dietary supplementation with omega-3 polyunsaturated fatty acids, and experimental studies have shown favorable effects of these fatty acids on mitochondrial function and dynamics (29), mainly through their action as PPARs ligands (30). Calanus oil is a novel marine oil, which is extracted from the marine crustacean, *Calanus finmarchicus*. It has a unique chemical composition and is rich in both poly- and monounsaturated fatty acids (31). We reported recently that dietary supplementation with Calanus oil restored metabolic flexibility in cardiac muscle from high fat-fed obese mice, and that this response was associated with improved mitochondrial respiration (17). However, it is important to understand how Calanus oil, as well as exenatide, could modify mitochondrial function during obesity also in major tissues like liver, skeletal muscle, and adipose tissue, which could have a differential response (32). In this regard, our focus has been to demonstrate how diet-induced obesity, and the super-imposed treatments with Calanus oil and exenatide, impact oxygen flux in the various tissues compared to corresponding flux values of lean control mice which were very different for liver, EDL, and WAT. These tissue differences in oxygen flux at the different respiratory states could be related to their respiratory capacity per mg of wet tissue, since it is well documented that mitochondrial density is higher in liver and skeletal muscle than in white adipose tissue.

The overall observation from the current study is that high-fat feeding leads to a marked reduction of mitochondrial respiration in adipose tissue during the three investigated states - LEAK, OXPHOS and ETS. This response was to some extent (not statistically significant) attenuated by exenatide treatment, but not with Calanus oil treatment. High-fat feeding had lesser effect on hepatic mitochondrial respiration than in adipose tissue, but exenatide treatment resulted in a significant increase in the various respiratory states in liver. Mitochondrial respiration in skeletal muscle was not significantly influenced by neither high-fat diet nor any of the treatments.

The general view is that uncoupling decreases mitochondrial ROS production, and therefore uncoupling may play a protective role by mitigating cellular ROS production (33). Moreover, a feedback loop between ROS and proton leak has been suggested, where proton leak decreases ROS generation and ROS, in turn, induces proton leak (34). Interestingly, although proton leak is regarded detrimental to ATP synthesis, it has been shown to be cytoprotective in cardiac muscle exposed to ischemia-reperfusion (35) and in diabetes (36). Based on absolute oxygen fluxes, it is therefore Tempting to suggest that the elevation in proton leak observed in liver tissue from exenatide-treated HFD mice was protective by preventing build-up of toxic lipid intermediates *via* increased fatty acid oxidation. However, when the relative contribution of LEAK to maximal respiratory capacity was determined, the differences among groups disappeared, indicating that absolute differences in LEAK were most probably related to a higher oxidative capacity than to a qualitative change in the oxidative phosphorylation performance. These observations reinforce the need to inquire a more in-depth analysis when addressing mitochondrial respirometry studies. Furthermore, going back to our results, it is likely that increased hepatic mitochondrial respiration in OXPHOS and ETS, prevented build-up of toxic lipid intermediates *via* increased fatty acid oxidation. This mechanism could contribute to the anti-obesogenic effect of exenatide by working as a sink, where mitochondrial oxidation drains fatty acids released from obese fat depots.

The finding that high-fat feeding led to reduced mitochondrial LEAK respiration in adipose tissue could be mediated by reductions in adipose tissue mitochondrial content due to the fact that those differences disappear when we analyze LEAK as a relative contribution to maximal respiratory capacity (37–39). Reduced LEAK respiration could, on the one hand, reflect reduced fatty acid oxidation causing intracellular build-up of toxic lipid intermediates, and on the other hand could imply increase mitochondrial efficiency. Exenatide treatment to some extent (not statistically significant) attenuated the HFD-induced decline in LEAK respiration and, in line with the current view, we suggest this might protect mitochondrial function from the lipid stress during high-fat feeding.

The explanation for the lack of any clear effect of Calanus oil on mitochondrial respiration function in this study is not obvious; except for relative contribution of LEAK to maximal respiratory capacity in liver, which could indicate a higher uncoupling capacity. As shown in previous studies (31, 40, 41), Calanus oil and exenatide have similar phenotypic effects (reduction of intra-abdominal adiposity and improved glucose tolerance). Thus, if reduced obesity and reduced fatty acid load are mechanisms behind the improvement in mitochondrial function following exenatide treatment, one would have expected the same response with Calanus oil treatment. However, the underlying biochemical and biophysical mechanisms by which omega-3 fatty acids in general influence mitochondrial structure and function are not clear (42, 43).

The study has some potential limitations that should be considered when assessing the results. Circulating levels of exenatide were not measured, but body weight evolution after minipump implantation indicated that exenatide was delivered properly. In addition, the significant effect of exenatide on liver mitochondrial bioenergetics should be supportive of an effective treatment. Similarly, we did not measure circulating levels of omega-3 fatty acids, since collection of blood samples gave low amounts of plasma - often hampered with hemolysis. However, results from a previous study (44) demonstrated increased accumulation of polyunsaturated omega-3 fatty acids (PUFA) in adipose tissue and liver following a similar HFD+Cal treatment as used in the present study, proving that Calanus-derived omega-3 fatty acids were indeed taken up from the diet into the circulation. This was also confirmed in a later study by the finding of a significant increase in the omega-3 index in mice given HFD supplemented with Calanus oil (17). The lack of an effect of Calanus oil supplementation on WAT, liver, and EDL muscle mitochondrial bioenergetics, in contrast to what we have previously found in heart (17), suggests that future experiments should address the duration, as well as dosage, of the Calanus oil supplementation on these tissues. Finally, citrate synthase data on WAT are not conclusive and we cannot clarify whether the differences observed in mitochondrial function are due to a significant reduction in mitochondrial mass. Nevertheless, existing literature and our recent publication showing a significant reduction in mitochondrial DNA content after the same length of a HFD-treatment in WAT of male mice pointed into this direction (25, 39).”

In conclusion, the GLP-1 analogue exenatide increased hepatic mitochondrial respiration in high-fat fed mice but had no clear beneficial effect in skeletal muscle of fat tissue. Calanus oil did not negatively affect respiratory activity in these tissues, which does not dismiss its potential as a dietary supplement, due to its previously reported benefits. Based on our current results, future studies could focus on the potential beneficial effects of exenatide and Calanus oil co-treatment.

## Data availability statement

The raw data supporting the conclusions of this article will be made available by the authors, without undue reservation.

## Ethics statement

All animal procedures were approved by the local ethics committee, Comitè Ètic d’Experimentació Animal at the Universitat de Barcelona and the Departament d’Agricultura, Ramaderia, Pesca, Alimentació i Medi Natural at the Generalitat de Catalunya, complying with current Spanish and European legislation. Authorized research project #9972, registered number by Generalitat de Catalunya: FUE-2018-00708549.

## Author contributions

Study design (TL and PG-R), funding (TL and PG-R), performing experiments (KJ, ND, PG-P, PCS and PG-R) data analysis (KJ and PG-R), manuscript preparation (KJ, ND, TL and PG-R) edition (ND, TL and PG-R). All authors contributed to the article and approved the submitted version.
